# How labelling of commercial infant food impacts parents’ beliefs about sugar content and related purchasing and feeding decisions: a scoping review

**DOI:** 10.1017/S1368980025100827

**Published:** 2025-08-13

**Authors:** Rana Conway, Tiffany Denning, Andrew Steptoe, Clare Llewellyn

**Affiliations:** Research Department of Behavioural Science and Health, University College London, London, UK

**Keywords:** Sugar, Infant food, Baby food, Parents, Food labelling

## Abstract

**Objective:**

To assess what is known about how the labelling of commercial infant food impacts parents’ beliefs about a product’s sugar content and their related purchasing and feeding decisions.

**Design:**

Mixed methods scoping review. Peer-reviewed studies were identified from six electronic databases, and grey literature was identified via Google, relevant websites, government reports and by contacting organisations. Searches were completed in May 2024 using a comprehensive search string incorporating keywords and indexed terms related to ‘parents’, ‘beliefs’, ‘sugar’ and ‘baby food labels’.

**Setting:**

Northern, Western and Southern Europe, North America, Australia and New Zealand.

**Participants:**

Parents and primary caregivers of children (≤ 37 months) or those specifically choosing commercial infant food for their children.

**Results:**

In total, 1123 records were screened, and seventeen were included for review, with all records published since 2015. Records reported on fifteen unique studies, including seven quantitative, seven qualitative and one mixed-methods study. Studies found that simply labelling products as suitable for babies elicited a trust that they were healthy, including not having a high sugar content. Interventions alerting parents to the sugar content of products were associated with less positive opinions or reduced intention to purchase. In eleven studies, parents described being drawn to products displaying labels such as ‘no added sugar’, which some perceived as meaning low sugar. In five studies, parents described sugar labelling as misleading, and/or they explicitly expressed a desire for clearer sugar labelling.

**Conclusions:**

Parents find the current labelling of commercial infant food misleading and desire clearer labelling to support informed purchasing and feeding decisions.

High sugar intakes can contribute to childhood obesity and dental caries, which present major public health challenges around the world^(1)^. In the UK, free sugars account for 10 % of calories consumed by children aged 1–3 years, which is double the national recommendation that free sugar intake should not exceed 5 % of total energy^(2)^. Free sugars include all added sugars, all sugars naturally present in fruit juices, purees and similar products in which the structure has been broken down and all sugars in drinks (except dairy-based drinks)^(2)^.

Many commercial infant foods and beverages (CIF) have a high sugar content but are marketed as healthy and appropriate for infants and young children up to the age of 36 months^(3–5)^. A survey of 3427 CIF across twenty-seven European countries found that half included the message ‘no added sugar’, yet 35 % of these contained free sugars^(6)^. Some parents report a general perception that CIF are healthy, which labelling such as this may contribute to^(7)^. ‘Health halo’ statements such as ‘natural’ and ‘organic’ and images of fruit also add to the belief that products are healthier than their nutrient profile would indicate^(8,9)^.

The WHO considers existing CIF composition and marketing regulations to be outdated and proposes new regulations, including clearer messaging relating to sugar content^(1,10)^. The UK Government has also made a commitment to support families to make healthier food choices. Leaving the European Union provides the UK greater flexibility for regulatory changes, such as making food labels clearer^(11)^. A range of front-of-pack (FoP) label formats is in use around the world to communicate energy and nutrient information, including sugar content. Mandatory policies for displaying traffic lights, nutrition scores, nutrition warnings or health warnings have been shown to be effective in changing children’s and adults’ purchasing behaviour towards healthier products and away from less healthy products, according to a systematic review and meta-analysis of 156 studies^(12)^. In the UK, the government recommends including multiple traffic light labels on most packaged foods to provide information at a glance and support consumers in making healthier food choices^(13)^. However, multiple traffic light labels are based on reference intakes for adults rather than infants or children, whose energy and nutrient requirements vary according to age^(13)^. The WHO proposes the use of sugar warning labels (SWL) on CIF as, in addition to helping parents recognise high-sugar products, they may incentivise the CIF industry to reformulate products and/or change product ranges^(1,10)^. A scoping review of experimental studies of nutrient warning labels on sugar-sweetened beverages (SSB) and ultra-processed foods found that SWL helped adults and children to identify high-sugar products and discouraged them from purchasing these products^(14)^. Both these reviews considered adults and children making food choices for themselves^(12,14)^. No reviews could be found on the impact of FoP labelling policies for sugar on the choices made by parents and other primary caregivers (referred to as ‘parents’ throughout for brevity) on behalf of their infants and young children.

A particular issue with CIF is the perception that because products are strictly regulated, they must be low in sugar, which is not always the case^(7)^. In addition, claims such as ‘no added sugar’ are common on CIF, as are claims about ‘natural sugar’, which is a term consumers may view more positively^(1,4,15)^. Also, fruit puree and concentrated fruit juice are commonly used to sweeten CIF, which are listed as such in the ingredients, although consumers are unaware that they contain high levels of free sugar^(1,4,15)^. It is important to bring together the available evidence to allow policymakers to better understand opportunities for policy levers to improve the labelling of CIF and identify any requirements for additional research.

The aim of this scoping review was to assess the published and unpublished evidence base to understand what is known about how the labelling of CIF impacts parents’ beliefs about sugar content and their related purchasing or feeding decisions. Specific review questions were: (i) what is known about how primary caregivers understand terms used on CIF to describe sugar? (ii) what is known about how primary caregivers might use SWL on CIF?

## Methods

A preliminary search of MEDLINE, PROSPERO and the Cochrane Database of Systematic Reviews was conducted, and no current or underway systematic reviews or scoping reviews on the topic were identified. This scoping review was informed by the Johanna Briggs Institute methodology^(16)^ and reported according to the Preferred Reporting Items for Systematic Reviews and Meta-analysis extension for Scoping Reviews (PRISMA-ScR, see online supplementary material, Supplemental File S1)^(17)^. The review protocol was preregistered with the Open Science Framework (doi: 10.17605/OSF.IO/S32CR).

### Definitions and eligibility criteria

For the purposes of this review, CIF was defined as any commercially prepared food or drink labelled as suitable for children ≤ 36 months of age, excluding commercial milk formula. ‘Sugar’ included mono- and disaccharides, such as glucose, fructose and sucrose, including those found in fruit. ‘Food labelling’ referred to any information, symbols or statements on packaging, including nutrition panels, ingredient lists, traffic light labels and warning labels. ‘Understanding’ encompassed parents’ interpretation and use of labels and other packaging information, including images, nutrient or health-related statements, which may influence their perceptions of a product’s sugar content.

Eligibility of studies was determined using the population, concept and context framework^(18)^. Studies were eligible for inclusion if they included (i) parents of children aged ≤ 37 months choosing foods/drinks for their child and/or parents’ choosing CIF; (ii) sugar; (iii) food labelling; (iv) parents’ understanding; (v) participants living in Northern, Western or Southern Europe, North America, Australia or New Zealand (to increase the generalisability of findings to the UK population); (vi) were written in English; and (vii) were accessible in full text.

Studies were excluded if they (i) involved a broader age group without presenting subgroup analysis for the target age group of ≤ 37 months, unless specifically focused on CIF; (ii) focused exclusively on commercial milk formula, product sales or parents’ perceptions of baby food marketing beyond packaging; (iii) were conducted in larger geographical regions without relevant subgroup analysis; and (iv) were published in a language other than English.

Quantitative, qualitative and mixed-method studies were included, as well as meta-analyses, opinion papers and non-peer-reviewed reports from government departments and third-sector organisations.

To avoid omitting important insights, the original inclusion criteria were expanded. The protocol originally specified inclusion of studies with parents of children aged 4–36 months, but this was expanded to parents of children ≤ 37 months. It was also planned that only studies of CIF would be included, but this was expanded to other foods or drinks if the study focused on the provision of these products to children aged ≤ 37 months. Updated inclusion criteria allowed the inclusion of eleven additional studies, four meeting age but not CIF criteria, six about CIF where age was outside the 4–36 month range and one about drinks which included parents of children aged ≤ 37 months.

### Information sources and search strategy

The search strategy was developed in consultation with a research librarian and designed to capture both published and unpublished studies. In May 2024, searches were conducted across six bibliographic databases: MEDLINE (Ovid), Embase (Ovid), PsycINFO (Ovid), CINAHL (Ebsco), Web of Science (Core Collection) and Cochrane Library, with no date restrictions. A comprehensive search string incorporating keywords and indexed terms related to ‘parents’, ‘beliefs’, ‘sugar’ and ‘baby food labels’ was initially developed for MEDLINE and then tailored to each database and information source (see online supplementary material, Supplemental File S2). The reference lists of all included sources were also screened to identify additional studies.

To access unpublished or non-academic reports, a thorough grey literature search strategy was implemented. This strategy involved three key approaches: (i) Google searches, (ii) targeted website searches and (iii) consultations with experts in the field. These complementary approaches minimised the risk of omitting relevant sources. Data from grey literature were evaluated for inclusion and extracted using the same eligibility criteria as for peer-reviewed papers. The complete grey literature strategy, search terms and screening process are detailed in online supplementary material, Supplemental File S3.

### Selection of sources of evidence

Identified citations were collated and uploaded into Covidence, where duplicates were removed. A rigorous two-step screening process was then undertaken. In the first stage, TD and CR independently reviewed titles and abstracts against the predefined inclusion and exclusion criteria, classifying each source as *include, exclude* or *unclear*. In the second stage, full texts of articles marked as *include* or *unclear* were retrieved and reassessed using the same criteria. Studies that met all the criteria were then included in the review.

**Fig. 1:**
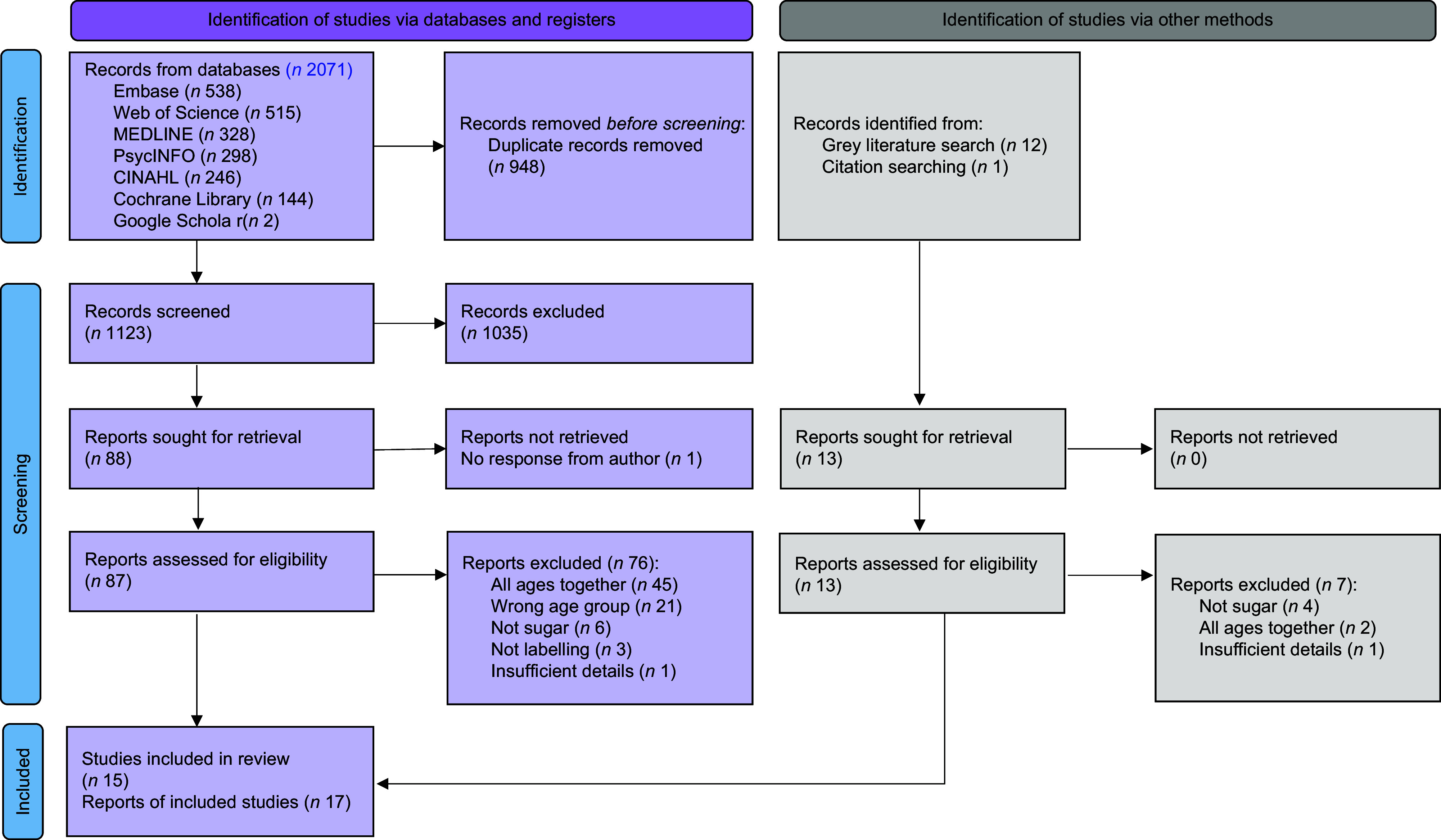
PRISMA flow diagram of search process.

To ensure unbiased selection, reviewers conducted their assessments independently and were blinded to each other. Consistency was maintained by TD and RC independently piloting the screening process with a random sample of twenty abstracts and ten full texts and discussing decision making before proceeding with the full literature review. Once both TD and RC had completed each stage of reviewing, disagreements were resolved through discussion. A summary of the selection process, including reasons for exclusion, is presented in Fig. 1.

### Data charting process

A data extraction form was developed in Microsoft Excel, adapted from the Johanna Briggs Institute methodology for scoping review guidelines. The form was piloted by TD and RC on five studies and modified before being applied to the remaining studies. Data charting was performed by TD and validated by RC, with any disagreements resolved through discussion.

The extracted data included the source/year of publication, year of study, country, aims, population/sample size, study design, funding source, summary of findings and any additional relevant insights. In the original data extraction form described in the protocol, it was intended that findings related to parents’ beliefs about sugar content in baby foods would be recorded separately from those related to feeding and purchasing decisions. However, when piloting the form, it was not possible to separate results in this way, so findings were documented in a single column. Study findings were then summarised for presentation and organised according to primary themes to provide a narrative summary. As is typical for scoping reviews, the quality of the studies was not evaluated.

## Results

### Summary of research results

The academic literature search yielded 2071 records from six databases, with 1123 remaining after deduplication. After screening the titles and abstracts, eighty-eight records were retrieved for full-text review. Despite efforts to contact authors, one full-text article could not be retrieved and hence was excluded. Of the eighty-seven remaining records, nine studies (reported in eleven papers) met the inclusion criteria. Citation searching identified one additional article, bringing the total to ten studies (across twelve papers).

The grey literature search employed three strategies: (1) a customised Google search with predefined parameters (reviewing the first ten pages for three keyword searches and three site-specific search strings), (2) targeted searches on thirty-four websites and (3) consultation with forty-nine experts, yielding twenty-four responses. Together, the three grey literature search strategies identified twelve sources, with five being retained after screening, including two identified through targeted website searches, two through Google and Google Scholar searches and one from contacting experts. Three of the five sources were charity reports, one was a government report and one was a master’s thesis.

Figure 1 shows the selection process for both peer-reviewed and grey literature, with the most common reasons for exclusion being the lack of subgroup analyses for the target age range or insufficient focus on sugar.

### Study characteristics

The source characteristics are summarised in Table 1. The review included fifteen studies published across seventeen papers published between 2015 and 2023. Geographically, five studies were conducted in the UK^(5,7,9,19–22)^, five in the USA^(23–27)^, four in Australia^(28–31)^ and one in Portugal^(32)^. The research designs varied: seven studies employed quantitative methods^(19,20,23,25,27,28,30)^, seven utilised qualitative approaches^(5,7,9,21,22,24,26,29,31)^ and one used mixed-methods^(32)^. All of the quantitative studies included cross-sectional surveys, and three also included a randomised controlled trial^(25,27,28)^. Qualitative data collection involved in-depth interviews (*n* 5), focus group discussions (*n* 2) and a netnographic analysis, which is a qualitative analysis of online communities. Two in-depth interview studies adopting a longitudinal approach. Sample sizes ranged from 83 to 1023 for quantitative studies and 11–227 for qualitative studies. Most studies were conducted online (*n* 10), with the remainder delivered in person (*n* 5).

**Table 1.** Characteristics of included studies (*n* 15)

Publication details	Year of study	Country	Aim	Population/sample size	Study design	Funding source
Action on Sugar, 2021^(19)^	2021	UK	To identify priorities in choosing snacks for infants and toddlers	Nationally representative sample of parents with children aged 6–36 m (*n* 1000)	Cross-sectional online survey	Impact on Urban Health
Action on Sugar, 2022^(20)^	2022	UK	To hear views on CIF	Nationally representative sample of parents with children aged 6–36 m (*n* 1000)	Cross-sectional online survey	Impact on Urban Health
Choi, 2022^(23)^	2019	USA	To investigate factors influencing the frequency of giving sweetened fruit-flavoured drinks, focusing on (i) perceptions of drink healthfulness, (ii) behavioural factors and (iii) socio-demographic factors	Random sample of caregivers with children aged 1 y (*n* 405) and 2 y (*n* 269) from online survey panel. Paper reports results for larger study	Cross-sectional online survey	Robert Wood Johnson Foundation
Fleming-Milici, 2022^(24)^	2019	USA	To explore (i) perceptions of sweetened fruit-flavoured drinks, (ii) potential misconceptions about their healthfulness and (iii) responses to factual information on ingredients and packaging claims	Convenience sample of parents with children aged 9–36 m (*n* 50) living in low to middle income neighbourhoods	Cross-sectional in-person focus groups	Robert Wood Johnson Foundation
Harris, 2022^(25)^	2021	USA	To test (i) the effects of two videos aimed to counteract common misperceptions about fruit drinks and (ii) the associated changes in intent to serve plain milk or water	Convenience sample of parents with children aged 8–37 m (*n* 600)	Cross-sectional online RCT	Robert Wood Johnson Foundation
City University of London, 2022–2024^(7,9,21)^	2020–2021	UK	To explore how social and environmental factors determine foods consumed over the first 18 m of life, focusing on differences by SEP	Purposive sample of parents with children aged 4–6 m (*n* 62), distributed across low (*n* 18), medium (*n* 22) and high (*n* 22) SEP. Follow-up when infants were 10–12 m (*n* 58) and 6–18 m (*n* 47)	Longitudinal online interviews	National Institute for Health and Social Care Research
Lovelace, 2015^(22)^	Not presented	UK	To explore feeding choices made by low-income families including socio-economic and environmental influences	Purposive sample of mothers with children aged 2–37 months (*n* 11), non-homeowners receiving income support and/or Healthy Start vouchers	Cross-sectional in-person interviews	Worcestershire Primary Care Trust and the public health department of the National Health Service Worcestershire
McCann, 2022^(28)^	2020	Australia	To investigate the relative impact of different regulated and unregulated claims on perceptions of the healthiness of an ultra-processed, discretionary toddler snack food	Convenience sample of parents with children aged 1–3 y (*n* 284)	Cross-sectional online survey & discrete choice experiment	University funds provided to the author as part of her PhD
Morel, 2019^(26)^	2017	USA	To examine beliefs, barriers and facilitators related to the avoidance of SSB and inform the development of culturally appropriate interventions to reduce consumption	Purposive sample of mothers with children < 2 y (*n* 16), from an initial cohort enrolled in the study’s quantitative phase (*n* 396)	Cross-sectional in-person interviews	Robert Wood Johnson Foundation
Poirier, 2021^(29)^	2011–2015	Australia	To explore the context in which Indigenous Australians experience oral health and identify barriers impeding parental efforts to establish oral health practices for their children	Purposive sample of parents with children aged 6–36 m (*n* 227), selected from participants enrolled in the South Australian Aboriginal Birth Cohort Study	Longitudinal in-person interviews	National Health and Medical Research Council of Australia and Channel 7 Children’s Research Foundation grant
Public Health England, 2019^(5)^	2018	UK	To fill knowledge gaps related to the Start4Life campaign and test example key messages	Mothers with children aged 7–12 m from middle and lower social grades (*n* 36)	Cross-sectional in-person interviews	Public Health England
Rhodes, 2022^(30)^	2021	Australia	To understand attitudes and understanding regarding the contents and regulations of CIF	Nationally representative sample of parents with children aged 0–4 y (*n* 1023)	Cross-sectional online survey	Royal Children’s Hospital Foundation
Rowan, 2022^(31)^	2019	Australia	To describe parental perceptions of the use of baby food pouches	Seventy-eight threads relating to squeezable baby food pouches, from parenting forums on six websites. Participant demographics not available	Online netnography	University of Otago summer studentship
Sousa, 2023^(32)^	2022	Portugal	To understand values and concerns regarding CIF to understand how companies can increase brand loyalty	Convenience sample of parents with children aged 6 m–3 y for qualitative study (*n* 15) and quantitative study (*n* 353)	Mixed methods; cross-sectional online focus groups; cross-sectional online survey	Universidade Nova de Lisboa
Taillie, 2022^(27)^	2020	USA	To determine the impact of a text or pictorial sugar warning label on snack choices	Convenience sample of parents with children aged 1 y (*n* 83). Child must have consumed at least one fruit drink within the last week. Paper reports results for larger study	Cross-sectional online RCT	Robert Wood Johnson Foundation

CIF, commercial infant food; m, months; RCT, randomised controlled trial; SEP, socio-economic position; SSB, sugar-sweetened beverage; y, years.

**Table 2.** Summary of findings relating to parents’ beliefs about sugar content and choice of foods for their infants and young children (*n* 15)

Publication details	Study design and methods	Summary of findings
Action on Sugar, 2021^(19)^	Cross-sectional online survey of parents; conducted via Censuswide	• 84 % buy ready-made sweet snacks; 92 % prefer natural sugar sources (e.g. fruit) • On-pack claims like ‘no added sugar’ (59 %), ‘1 of your five a day’ (51 %) and ‘no artificial preservatives’ (39 %) reported to influence purchases • 69 % check sugar content on packaging for commercial baby and toddler sweet snacks • 50 % believe ready-made CIF snacks are as healthy as homemade; 15 % think they are healthier
Action on Sugar, 2022^(20)^	Cross-sectional online survey of parents; conducted via Censuswide	• Top reasons reported for buying sugary toddler breakfast products: convenience, child’s preference, trusted brand, no added sugar, more vitamins/minerals • 62 % think fruit juice should be limited for dental health; 65 % are concerned about sugar levels in toddler breakfast items • 87 % would find clear labelling of added sugars on front packaging useful • 91 % support government action to ensure baby aisle products meet National Health Service nutritional guidelines
Choi, 2022^(23)^	Cross-sectional online survey; reasons for serving fruit SSB or flavoured water to children ≤ 2 years and perceived importance of product attributes (healthfulness, product claims and other characteristics)	• SSB were perceived as unhealthy by caregivers of children aged 1 year and 2 years (mean (sd)) 3·5 (2·0) and 3·4 (1·6), respectively; on a scale from 1 = very unhealthy, 10 = very healthy • Importance of product features (mean (sd) on scale from 1 = not at all important to 7 = extremely important): caregivers of children aged 1 year: natural^*^ 4·8 (1·5), 100 % juice 5·5 (1·5), good source of vitamin C 5·5 (1·4), no/less sugar 5·3 (1·4), low in calories 4·1 (1·9); caregivers of children aged 2 years: natural^*^ 4·7 (1·5), 100 % juice 5·4 (1·5), good source of vitamin C 5·2 (1·4), no/less sugar 5·0 (1·5), low in calories 3·7 (1·9)
Fleming-Milici, 2022^(24)^	Cross-sectional in-person focus groups; discussed concept sheets with facts about SSB (focusing on ingredients, claims, marketing messages) and expert recommendation sheets (traffic light system of 3 drink categories to encourage plain milk/water, limited 100 % juice, avoid SSB)	• Labelling perceived as deceptive - minimising sugar content while emphasising health benefits • Claims (e.g. 100 % fruit juice, all natural, less sugar) hide sugar content and difficult to identifying sugar and NNS in ingredient lists • Targeted marketing to parents implies products are healthy for children through claims like 100 % juice • SSB advertised as juice boxes, so assumed to be healthy. Limited time to differentiate between products while shopping
Harris, 2022^(25)^	Cross-sectional online RCT; questionnaire on child’s drink consumption; randomised into two groups: group 1 viewed counter-marketing videos on SSB (showing unhealthy ingredients, recommending water/milk); group 2 viewed control videos on screentime and co-viewing. Measured positive attitudes, behavioural intentions and normative beliefs for serving SSB *v*. water/milk	• 50 % of caregivers had served SSB to their child in the past month • Counter-marketing videos reduced caregivers’ overall positive attitudes about SSB by a mean of 0·92 (% CI = 0·52, 1·32) on a 10-point scale from harmful to beneficial • Counter-marketing videos reduced intentions to serve SSB in the next month by a mean of 0·50 (95 % CI = 0·22, 0·77) on a 6-point scale from strongly disagree to strongly agree
City University of London, 2022–2024^(7,9,21)^	Longitudinal 1–2–1 online interviews; T1 at child age 4–6 m, T2 at 10–12 m, T3 at 16–18 m; interviews explored (1) infant feeding experiences, (2) reasons for feeding methods and (3) personal, social, cultural and economic influences on feeding. Included photo elicitation exercise where participants shared photos depicting factors influencing infant feeding decisions	• Over the 18 m, parents gradually relaxed about sugary snacks, for example, introducing ‘adult’ snacks like biscuits, especially in households with older siblings • Receiving mixed messages about weaning from different sources caused confusion, leading many to rely on branded products for guidance • Trust in CIF was influenced by packaging claims like ‘no nasties’ and ‘organic’, with parents seeking simple, clear ingredient lists. • Packaged snacks chosen for non-nutritional reasons, focus on avoiding harmful ingredients rather than seeking positive nutritional value • Baby aisle products were generally trusted as healthy, nutritious and age appropriate
Lovelace, 2015^(22)^	Cross-sectional 1–2–1 in-person interviews; exploring eating behaviours, family food budget and choices; grounded theory approach	• Parents trusted CIF, assuming products like baby biscuits and rusks were suitable without checking sugar or fat content • Many avoided obvious sweet foods like chocolate but gave baby snacks without worrying about sugar levels • Confusion about what was healthy, many relied on labels like ‘low sugar’ without checking the full ingredient list • Many reported choosing familiar products and not regularly checking labels for details like salt or sugar
McCann, 2022^(28)^	Cross-sectional online survey with discrete choice experiment; assessed how claims influence consumer perceptions of an ultra-processed toddler snack bar. Participants chose the ‘most and least healthy’ product from three options with varying claims	• Products with nutrition claims perceived as healthier. Compared with products without a claim, participants were 14× more likely to perceive a product with the claim ‘no added sugar, no added salt’ as most healthy (OR 13·71; CI: 10·93, 17·22, *P* < 0·001) • The combination of ‘no added sugar, no added salt’ and ‘no added preservatives, colours or flavours’ had the strongest influence on perceptions of healthiness
Morel, 2019^(26)^	Cross-sectional 1–2–1 in-person interviews exploring (1) perceptions, barriers and facilitators of avoiding SSB, (2) acceptability of intervention messages and (3) perceived impact of interventions on SSB consumption.	• Many confused about healthy beverages, often misidentifying SSB as healthy • Reliance on branding/packaging over nutrition labels to assess sugar content and shocked by high sugar content of products perceived to be healthy (e.g. sports drinks and 100 % juice) • Using images of health consequences of SSB consumption considered potentially effective for reducing consumption as parents want to promote infant health • Preference for labels with specific guidelines on frequency/volume over vague terms like ‘rarely’, and belief that showing sugar content would empower better choices • Desire for clear, visual messaging delivered through various formats (e.g. posters, videos, apps) and repeated in different settings, including at point of sale
Poirier, 2021^(29)^	Longitudinal in-person interviews; T1 baseline during pregnancy, T2 at child age 6 m, T3 at 12 m, T4 at 18 m. At T2, the importance of non-cariogenic foods and drinks was emphasised. Project nested within RCT of an Early Childhood Caries prevention intervention designed and conducted in partnership with Indigenous families and communities	• Nutrition knowledge was limited and misleading. FoP claims contributed to misconceptions; for example, some believed certain products (e.g. Kinder Surprise) were healthier due to marketing claims (e.g. ‘more milk content’) • Reliance on marketing claims like ‘pure fruit, no added sugar’ when choosing CIF, without realising the actual sugar content • When ranking CIF by sugar content, almost all parents who didn’t read nutrition labels, based their decisions on claims, specifically ‘no added sugar’; shocked by the high sugar content in baby CIF and called for clearer SWL on packaging • Lack of time/energy to read detailed information • Many felt overwhelmed by the prevalence of sugar hidden in everyday foods
Public Health England, 2019^(5)^	Cross-sectional interviews either 1–2–1 or friendship pairs. Explored sources of information, knowledge, attitudes and experiences of weaning including drivers of choice	• Participants trusted food manufacturers to know what is best for babies • CIF jars, pouches and snacks assumed to be healthy and appropriate for babies’ needs and therefore would not contain too much sugar/salt Labelling linking to fruit and vegetables suggested benefits and good nutritional content • Use of ‘organic’, ‘preservative free’, ‘no added sugar, etc.’, and headline vegetable ingredients/borrowing real food names (e.g. carrot sticks), especially on premium brands, suggested these were healthy products. Many assumed foods labelled ‘no added sugar/salt’ were low in sugar/salt and therefore appropriate • Believed to be unnecessary to examine labels if labelling suggested that products were healthy • Low awareness that CIF can be high in sugar
Rhodes, 2022^(30)^	Cross-sectional online survey regarding experiences, behaviours, attitudes and beliefs about purchasing and serving CIF	• 73 % chose CIF because they believed products were healthy choice and 30 % believed CIF were as healthy as food made at home • Majority reported claims on CIF were very or extremely likely to influence their choices, including ‘natural’ (93 %), ‘made with fruit and veg’ (93 %), ‘no added sugar’ (91 %), ‘sweetened with fruit’ (87 %) • 53 % falsely believed CIF content was regulated by government to ensure it provided good nutrition for children • Most supported introducing laws to regulate the content and marketing of CIF (90 %), the amount of harmful sugar in CIF (92 %) and the words, images and claims on CIF (90 %)
Rowan, 2022^(31)^	Netnographic study of six using publicly available online parenting forums. Seventy-eight threads relating to squeezable baby food pouches were analysed thematically. Counts of themes used for content analysis	When examining parental attitudes regarding the benefits and concerns of pouch use, the theme ‘High sugar’ was referenced sixty-five times, second only to ‘low nutritional value’, which was referenced seventy-one times
Sousa, 2023^(32)^	Cross-sectional online focus groups followed by online survey to confirm and generalise qualitative findings	Qualitative interview: • As babies grew older, mothers felt less pressure and increasingly opted for commercial products as convenience outweighed perceived risks • Primary concern was ingredients; shorter lists without added sugars or ingredients like palm oil, sweeteners and preservatives were preferred • Sugar is seen as necessary for healthy development; therefore, no desire to ban it but favoured delaying introduction and then give appropriate types in moderation. Strong desire to avoid added sugars, especially in the early months • Wary of sugar substitutes and concern that products labelled ‘no added sugar’ may contain harmful ingredients Quantitative survey: mothers agreed that they preferred CIF without added sugar, mean score 6·4 on a scale from 1 (totally disagree) to 7 (totally agree)
Taillie, 2022^(27)^	Cross-sectional online experiment. Participants choose one of two packs of granola bites (without an added label or with an added label). They were randomised to view one of three added labels (barcode control, text-only SWL, pictorial SWL)	Results for parents of a 1 year old (no statistical analysis presented as these were a subset of participants): • 81 % chose the product with a barcode control, when presented with no added label or barcode control • 67 % chose the product with a SWL, when presented with no added label or a SWL (either text-only or pictorial)

CIF, commercial infant food; FoP, front of pack; m, months; NNS, non-nutritive sweeteners; RCT, randomised controlled trial; SWL, sugar warning label; T, timepoint.

* Natural feature includes ‘All natural’, ‘Organic’, ‘No HFCS’, ‘No artificial ingredients’ and ‘Non-GMO’.

One study exclusively recruited mothers, while others sought to recruit all parents or primary caregivers although their samples also primarily comprised mothers. Thirteen studies recruited parents of children ≤ 37 months, including three that included only parents with children < 24 months. The other two studies focused exclusively on CIF labelling but included parents with children ≤ 3 years and ≤ 4 years^(28,30)^. Three studies included parents of older children too; therefore, only findings for younger children were extracted to ensure alignment with our target population^(23,26,27)^. Some studies specifically recruited participants from low-income neighbourhoods^(24,26)^, non-homeowners receiving income support or healthy start vouchers^(22)^ or those enrolled in the Special Supplemental Nutrition Program for Women, Infants, and Children^(26)^. One study required participants to have a child who had consumed at least one fruit drink in the past week for eligibility^(27)^. Four studies focused specifically on SSB^(23–26)^, which they defined as drinks with added sugar, with one study also including drinks with only non-nutritive sweeteners^(25)^. Although definitions of SSB included products such as fizzy drinks and flavoured water, the focus was generally on fruit-flavoured drinks, excluding 100 % juice.

### Results of individual sources of evidence and synthesis of results

Key findings from each study are summarised in Table 2.

#### Impact of labelling products as suitable for babies

Studies exploring general beliefs about CIF (*n* 8) found that most parents assumed products labelled as suitable for babies were inherently healthy and appropriate^(5,7,9,19–22,29–31)^. Even though parents avoided giving foods they regarded as high in sugar, their trust in CIF meant they didn’t perceive sugar to be something they needed to consider when choosing from the baby food aisle. Parents from low-income families in the UK described offering ‘baby biscuits’ and rusks in the belief that they weren’t high-sugar products^(22)^. One participant noted, ‘I thought because they were baby foods, like baby stuff, they’d be careful about what sugar and stuff they put in them’ and another echoed this sentiment ‘With things like chocolate … we try to avoid that because obviously it’s gonna rot his teeth … we usually give him a couple of rusks or crisps … things like that’^(22)^.

In an online survey of 1000 UK parents by Action on Sugar, brand trust was given as a key reason for choosing to buy CIF breakfast products^(20)^. The impact of this trust was elucidated in a qualitative study, where parents described turning to brands for feeding guidance, one mother said ‘I quite like the Ella’s Kitchen … they do so many different flavours. I wasn’t put off by the ingredients as such. I find the organic pouches have got lovely ingredients in them. I don’t worry. I don’t think, oh, there’s too much sugar in this or there’s an E number or anything like that’^(7)^. Netnographic analysis of online parenting forums in Australia also suggests parents generally have a positive view of CIF pouches and view them as healthy^(31)^. A poll of 1023 Australian parents specifically explored perceptions around regulation and found that 53 % falsely believed that CIF were regulated by the government to ensure they provided good nutrition for babies and toddlers and 41 % believed that CIF must be healthy or the government wouldn’t allow them to be sold^(30)^.

#### Strategies to raise awareness of sugar content

Three studies conducted in the USA employed methods to increase awareness of the sugar content of products, including a randomised online experiment with SWL^(27)^, a randomised online experiment with counter-marketing videos^(25)^ and a study where information sheets were discussed in focus groups^(24)^. The first study involved a virtual shopping task, in which parents chose one of two snacks (with or without an ‘added label’). The ‘added labels’ were either a barcode control, text-only SWL or pictorial SWL. Among parents of 1-year-olds (*n* 83), 33 % chose a barcode control snack, while only 19 % selected the same item when an SWL was shown^(27)^. Statistical analysis was not performed for this subgroup of the larger sample of parents of 1 to 5-year-olds. In the second intervention study, parents viewed counter-marketing videos about SSB (described as sweetened fruit drinks) or videos about screen time (control group)^(25)^. Videos about SSB highlighted that closer examination of labels showed high sugar content and the presence of ingredients that were forms of added sugar or non-nutritive sweeteners and recommended only giving water and plain milk to toddlers^(25)^. After watching the videos, parents reported a reduced intention to serve SSB to their children, and they had less positive attitudes towards them, seeing them as less beneficial, convenient and good value. However, for parents who had recently served SSB, the videos did not significantly affect their intention to serve SSB. In the third intervention, parents in in-person focus groups reviewed ‘expert recommendation sheets’ and ‘concept sheets’ related to SSB (described as sugar-sweetened fruit-flavoured drinks)^(24)^. The expert sheet used a traffic light system to guide parents’ choices (green for plain milk and water, orange for 100 % juice and red for SSB), while the concept sheets addressed how to identify sugars in ingredient lists, how to find the total sugar content of a product in the nutrition facts panel and the meaning of common marketing claims. Over the course of focus group discussions, parents’ attitudes towards fruit SSB became less positive, and they became less accepting of the way they are marketed^(24)^.

In addition to the three intervention studies, a fourth study involved discussions with parents (*n* 9, USA) to explore the potential of using various strategies to promote avoidance of SSB^(26)^. Parents supported the use of illustrations of sugar content at the point of purchase and believed messages focusing on negative health consequences for children would be effective in changing parents’ feeding behaviour^(26)^.

#### Role of marketing claims

In eleven studies, parents reported being attracted to products labelled as having no added sugar, less sugar or only natural sugar^(5,7,19,20,22–24,28–30,32)^. Research from Public Health England described labels such as ‘no added sugar’, as well as more general claims such as ‘organic’ and ‘preservative free’ as reinforcing the parents’ trust in CIF brands to know what is best for babies and the perception that products were healthy^(5)^. Evidence from Australia also points to the persuasive power of specific claims, with the majority of parents reporting that claims on packs were very or extremely likely to influence their choices, including ‘no added sugar’ (91 %) and ‘sweetened with fruit’ (87 %)^(30)^. Similarly, an online survey of UK parents found that the belief that a product contained ‘naturally occurring sugars only’ was among the top five reasons given for choosing sugary toddler breakfast foods^(20)^. An online survey in Australia also found parents relied on claims such as ‘pure fruit, no added sugar’, without realising the total sugar content of products, while the claim ‘no added sugar’ was interpreted as meaning a product was low in total sugar^(29)^. In longitudinal interviews too, parents in Australia, particularly those reporting not reading nutrition labels, described ranking the healthiness of baby foods on claims such as ‘no added sugar’^(29)^.

Misconceptions conferred by claims relating to added and natural sugar were further reinforced by the perception that ingredients were presented clearly, honestly and devoid of jargon, making parents feel confident in their choices^(7)^. One parent, described as lower socio-economic position stated, ‘Basically they’re [Ella’s Kitchen are] easy to read ingredients wise. It is exactly what it says on the packet. There is no added salt, sugar, sweeteners. And obviously with me having to be careful with what I’m feeding [baby] they’re just very clear-cut and there’s no nonsense, and there are no scientific big words to try and decipher’^(7)^.

#### Demand for clearer labelling

In all fifteen studies, there was an indication that parents were unaware of the high level of sugar in some CIF. In two quantitative studies and three qualitative studies, parents explicitly said that on-pack messaging was confusing or deceptive and/or that they wanted clearer sugar labelling. In a poll of parents of 6- to 36-month-olds (*n* 1000, UK), 87 % said they would find it useful if the amount of sugars added to baby and infant food and drinks were displayed clearly on the FoP^(20)^. Similar views were expressed by parents in Australia (*n* 1023), with the vast majority supporting laws to regulate the content and marketing of CIF (90 %), the amount of harmful sugar in CIF (92 %) and words, images and claims on packs (90 %)^(30)^.

In qualitative studies, after being alerted to back-of-pack information showing sugar content and sugar ingredients, parents in the USA described labels as highlighting fruit ingredients but hiding sugar, which they considered confusing, deceptive and misleading^(24)^. Some parents went on to say that even if ingredient lists were more prominent, they might struggle to recognise which ingredients contain sugars^(24)^. Some parents expressed anger over deceptive marketing, with one parent saying, ‘Marketing people know how to market – all natural, oh, 100 % of vitamin C, oh that’s cool. Yeah, but also 100 % of your sugar for the day. It didn’t mention that on the front’^(24)^. In another study conducted in the USA, parents said they wanted clear information on sugar content, for example, expressed in terms of teaspoons, to empower them to compare products and make informed choices^(26)^.

In line with parents in the USA, those in Australia were annoyed to discover that products labelled ‘no added sugar’ sometimes had the highest sugar content^(29)^. One parent remarked, ‘[That]’s disgusting. They shouldn’t be able to make things like that, they should have a big sign on the front, [with] “high sugar content,” like they do with smoking’^(29)^. Parents also felt overwhelmed by the pervasive presence of sugar in foods and the difficulty in identifying truly healthy options. They emphasised the need for simple FoP labels to help balance health concerns with the demands of parenting, especially given time constraints. As one parent noted, ‘He’s very full-on, so [I] just feed him and then do my washing… It’s just too full-on to be able to [read nutrition labels]. If it had on the front of the packaging how many tablespoons of sugar, I’d probably think a second about getting him certain things, but it doesn’t. People don’t have time to read that. The mums that I know… they just go for what’s easy’^(29)^.

## Discussion

This review presents a comprehensive synthesis of the available evidence regarding how the labelling of CIF impacts parents’ beliefs about sugar content and related purchasing and feeding decisions. Fifteen studies were identified, including eight that explored parents’ general beliefs about CIF, revealing an implicit trust that products labelled as baby foods were healthy. The impact of SWL was only assessed in one study, but this study, along with two other interventions that raised parental awareness of sugar content, resulted in less favourable opinions or reduced intentions to purchase. Claims such as ‘no added sugar’ distracted parents from recognising CIF containing high levels of sugar and parents reported finding such labels confusing or they explicitly expressed a desire for clearer labelling. None of the studies specifically explored understanding of terms such as added sugar, natural sugar or free sugar.

The widespread trust that products labelled as suitable for babies are nutritionally appropriate is incongruent with evidence showing high levels of sugar in many CIF. Analysis of 2632 CIF products from ten European countries found that on average, one-third of the calories in CIF came from total sugars, and for most product categories, sugar contributed more than 10 % of calories^(3)^. As many CIF are pureed or sweetened with fruit juice, the sugars are largely free sugar, and therefore, the sugar content is inappropriately high given the recommendation that free sugar intake should not exceed 5 % of calories^(2,5)^. The perception that CIF are tightly regulated provided parents with confidence that products were low in sugar and meant they didn’t feel the need to scrutinise labels further. However, while legislation in place in the UK, as in many other countries, limits the amount of certain macro- and micro-nutrients in CIF, for most CIF, there is no limit on the total sugar content^(33)^. The WHO’s Nutrient and Promotion Profile Model (NPPM) proposes setting a maximum limit of 15 % energy from total sugar in CIF meals and snacks^(10)^. Parents appear to support such measures – a survey for Action on Sugar found 91 % of 1000 parents of children aged 6 to 36 months old in the UK wanted government action to ensure all food and drinks available in the baby food aisle are nutritionally appropriate according to National Health Service recommendations^(20)^. This review didn’t expressly aim to assess support for sugar content regulation, but as parents perceived sugar limits were already in place or expressed a desire for such regulations, this supports WHO calls for action.

One of the specific questions this review aimed to address was to understand what is known about how parents might use SWL on CIF. Only one study tested this, finding that fewer parents chose a toddler snack with a SWL, although statistical analysis was not presented for the subgroup of participants meeting the review’s inclusion criteria^(27)^. Results from the full sample, which included parents of children aged 1–5 years (*n* 2219), found that participants exposed to a text or pictorial SWL were less likely to select the labelled snack than those in the barcode control group (21, 18 and 34 % respectively; *P* < 0·001 for both comparisons of SWL to control)^(27)^. The impact of text and pictorial SWL was similar despite the authors having anticipated that the pictorial SWL would elicit greater attention and have a larger effect^(27)^. Three-quarters of parents in the complete sample also reported learning something new from the SWL, compared with one-quarter of those viewing the barcode control, providing further evidence of current misperceptions surrounding sugar content^(27)^. The positive impact on children’s diets of raising parental awareness of sugar was also demonstrated by the studies using counter-marketing videos and information sheets; however, such strategies are unlikely to have as wide a reach as FoP labelling regulations^(34)^. Parents receiving information sheets felt that marketing and packaging of SSB misled them regarding fruit juice content, which was commonly regarded as a healthy, sugar-free option^(24)^. This perception was due at least in part to interventions highlighting the sugar content of SSB without indicating that 100 % juice also contained sugar^(24)^. Findings from these intervention studies, together with more robust evidence regarding adults and children choosing products for themselves, suggest that any form of intervention is likely to impact parents’ perceptions of sugar content and may positively alter feeding decisions. However, given the dearth of evidence specifically regarding SWL on CIF, particularly regarding what alternative foods parents might choose if SWL were added to CIF, further research is needed about the potential benefits and inadvertent harms to inform policy development.

The majority of studies (eleven of fifteen) found that parents were attracted to products labelled ‘no added sugar’ or similar. The misleading nature of such claims was highlighted in several of the studies reviewed; for example, Poirier *et al.* found parents in Australia (*n* 200) used such claims to rank CIF by sugar content, and they were shocked to discover products’ actual sugar content^(29)^. None of the studies expressly sought to explore parents’ understanding of the terms added sugar or natural sugar, which was a specific question for this scoping review. A rapid review of literature related to general consumer understanding of terms such as added sugar (seventeen studies) found that ‘natural sugars’ were perceived as healthier than ‘added sugar’, and only 22–65 % of consumers identified fruit juice on an ingredient list as being added sugar^(15)^. As such claims are common, for example, ‘no added sugar’ or ‘less sugar’ was displayed on 58 % of 724 CIF examined in the UK, a clearer understanding of parents’ perceptions of sugar terminology is needed^(4)^. This extends to ingredient lists as ‘no added sugar’ claims are common across Europe on CIF containing pureed fruit or concentrated fruit juice despite these ingredients being high in total and free sugar^(3)^. As CIF display multiple claims (a median of 5 per product in the UK), parents’ perception of sugar terminology cannot be considered in isolation^(35)^. This review shows that claims such as ‘natural’, ‘organic’ or ‘no junk’ contribute to an overall assessment of products as ‘healthy’ and distract parents from scrutinising labels more closely^(4,7)^. Interestingly, parents’ increasing prioritisation of products perceived as ‘natural’, and their desire for ‘clean labels’ and limiting sugar is framed as an ‘opportunity’ in market research reports, rather than an area where clearer labels are needed to facilitate informed decision making^(36,37)^. The WHO argues that all marketing claims should be prohibited on CIF as they mislead parents and undermine public health messaging^(10)^. When alerted to the high sugar content of some CIF, parents felt current labelling was misleading and deceptive, and they expressed a demand for clearer FoP sugar labelling.

Strengths of this review included using six electronic databases alongside a thorough grey literature search to identify both quantitative and qualitative evidence and allow for a comprehensive account of current evidence. Potential limitations of the review were the exclusion of articles not written in English and the reliance on behavioural measures, such as intentions to purchase or serve, which may not be representative of real-world behaviours.

In conclusion, most parents trusted that products labelled as suitable for babies were healthy and appropriately low in sugar. This perception was reinforced by health halo messaging, and when parents were alerted to the high sugar content of some products, they felt current labelling was deceptive and/or expressed a strong desire for clearer labels. Parents’ understanding of terms such as added sugar was unclear, as was the impact of introducing SWL. However, results show the current lack of regulation leaves parents vulnerable to making underinformed choices for their children. While results support calls for legislation to make CIF labelling clearer, to support parents to reduce the free sugar intake of infants and young children, they highlight the need for policy research to examine more carefully the relative benefits and harms of introducing SWL on CIF.

## Supporting information

Conway et al. supplementary material 1 Conway et al. supplementary material

Conway et al. supplementary material 2 Conway et al. supplementary material

## Supplementary material

For supplementary material accompanying this paper, visit https://doi.org/10.1017/S1368980025100827.

## References

[1] World Health Organization (2019) Ending Inappropriate Promotion of Commercially Available Complementary Foods for Infants and Young Children between 6 and 36 Months in Europe. WHO European Region. https://www.who.int/europe/publications/i/item/WHO-EURO-2019-3590-43349-60813 (accessed October 2021).

[2] Scientific Advisory Committee on Nutrition (2023) Feeding Young Children Aged 1 to 5 Years. London. https://assets.publishing.service.gov.uk/government/uploads/system/uploads/attachment_data/file/1167077/SACN-Feeding-young-children-aged-1-to-5-full-report.pdf (accessed July 2023). 10.1017/S0029665126102250 41748544

[3] Hutchinson J , Rippin H , Threapleton D et al. (2021) High sugar content of European commercial baby foods and proposed updates to existing recommendations. Matern Child Nutr 17, 1–14. 10.1111/mcn.13020 PMC7729710 32862552

[4] Garcia AL , Menon R & Parrett A (2022) Extensive use of on-pack promotional claims on commercial baby foods in the UK. Arch Dis Child 107, 606–611. 35228205 10.1136/archdischild-2021-322851

[5] Public Health England (2019) Foods and Drinks Aimed at Infants and Young Children: Evidence and Opportunities for Action. https://assets.publishing.service.gov.uk/government/uploads/system/uploads/attachment_data/file/812204/Foods_and_drinks_aimed_at_infants_and_young_children_June_2019.pdf (accessed May 2020).

[6] Grammatikaki E , Wollgast J & Caldeira S (2021) High levels of nutrients of concern in baby foods available in Europe that contain sugar-contributing ingredients or are ultra-processed. Nutrients 13, 3105. 34578982 10.3390/nu13093105 PMC8466462

[7] Isaacs A , Neve K & Hawkes C (2022) Why do parents use packaged infant foods when starting complementary feeding? Findings from phase one of a longitudinal qualitative study. BMC Public Health 22, 2328. 36510175 10.1186/s12889-022-14637-0 PMC9744586

[8] Brunacci KA , Salmon L , McCann J et al. (2023) The big squeeze: a product content and labelling analysis of ready-to-use complementary infant food pouches in Australia. BMC Public Health 23, 656. 37024884 10.1186/s12889-023-15492-3 PMC10077707

[9] Gallagher-Squires C , Isaacs A , Reynolds C et al. (2023). Snacking practices from infancy to adolescence: parental perspectives from longitudinal lived experience research in England. Proc Nutr Soc (Published online) 1–9. doi: 10.1017/S0029665123003592 37759428

[10] WHO Regional Office for Europe (2022) Nutrient and Promotion Profile Model: Supporting Appropriate Promotion of Food Products for Infants and Young Children 6–36 Months in the WHO European Region. https://www.who.int/europe/publications/i/item/WHO-EURO-2022-6681-46447-67287 (accessed August 2023).

[11] Department of Health (2016) Childhood Obesity: A Plan for Action. https://www.gov.uk/government/publications/childhood-obesity-a-plan-for-action (accessed May 2021).

[12] Song J , Brown MK , Tan M et al. (2021) Impact of color-coded and warning nutrition labelling schemes: a systematic review and network meta-analysis. PLoS Med 18, e1003765. 34610024 10.1371/journal.pmed.1003765 PMC8491916

[13] Department of Health and Social Care (2020) Building on the Success of Front-of-Pack Nutrition Labelling in the UK: A Public Consultation. https://assets.publishing.service.gov.uk/government/uploads/system/uploads/attachment_data/file/905096/front-of-pack-labelling-consultation-document-english.pdf (accessed November 2024).

[14] Taillie LS , Hall MG , Popkin BM et al. (2020) Experimental studies of front-of-package nutrient warning labels on sugar-sweetened beverages and ultra-processed foods: a scoping review. Nutrients 12, 569. 32098363 10.3390/nu12020569 PMC7071470

[15] Food Standards Australia New Zealand (2022) Rapid Systematic Literature Review for P1058-Nutrition Labelling About Added Sugars. https://www.foodstandards.gov.au/sites/default/files/2023-12/P1058 %20Literature%20Review.pdf (accessed November 2024).

[16] Peters MDJ , Godfrey C , McInerney P et al. (2020) Chapter 11: Scoping Reviews. JBI Manual for Evidence Synthesis. JBI. https://jbi-global-wiki.refined.site/space/MANUAL/355863557/Previous+versions?attachment=%2Fdownload%2Fattachments%2F355863557 %2FJBI_Reviewers_Manual_2020June.pdf&type=application%2Fpdf&filename=JBI_Reviewers_Manual_2020June.pdf#page=406 (accessed February 2024).

[17] Tricco AC , Lillie E , Zarin W et al. (2018) PRISMA extension for scoping reviews (PRISMA-ScR): checklist and explanation. Ann Intern Med 169, 467–473. 30178033 10.7326/M18-0850

[18] Peters MDJ , Godfrey C , McInerney P et al. (2022) Best practice guidance and reporting items for the development of scoping review protocols. JBI Evid Synth 20, 953–968. https://journals.lww.com/jbisrir/fulltext/2022/04000/best_practice_guidance_and_reporting_items_for_the.3.aspx (accessed February 2024). 35102103 10.11124/JBIES-21-00242

[19] Action on Sugar (2021) The Sugar Content of Baby and Toddler Sweet Snacks and the Health Halo that Surrounds Them. https://www.actiononsugar.org/media/actiononsugar/Action-on-Sugar-Baby-&-Toddler-Sweet-Snacks-Report.pdf (accessed August 2024).

[20] Action on Sugar (2022) Awareness Week Report – Baby and Toddler Breakfasts. https://www.actiononsugar.org/media/actiononsugar/sugar-awareness-week/2022/Sugar-Awareness-Week-Report---Baby-&-Toddler-Breakfasts.pdf (accessed August 2024).

[21] Neve KL , Coleman P , Hawkes C et al. (2024) What shapes parental feeding decisions over the first 18 months of parenting: insights into drivers towards commercial and home-prepared foods among different socioeconomic groups in the UK. Appetite 196, 107260. 38403201 10.1016/j.appet.2024.107260

[22] Lovelace S & Rabiee-Khan F (2015) Food choices made by low-income households when feeding their pre-school children: a qualitative study. Matern Child Nutr 11, 870–881. 23320519 10.1111/mcn.12028 PMC6860335

[23] Choi YY , Jensen ML , Fleming-Milici F et al. (2022) Caregivers’ provision of sweetened fruit-flavoured drinks to young children: importance of perceived product attributes and differences by socio-demographic and behavioural characteristics. Public Health Nutr 25, 2308–2316. 35440350 10.1017/S1368980022000751 PMC9991680

[24] Fleming-Milici F , Phaneuf L & Harris JL (2022) Marketing of sugar-sweetened children’s drinks and parents’ misperceptions about benefits for young children. Matern Child Nutr 18, e13338. 35199914 10.1111/mcn.13338 PMC9218304

[25] Harris JL , Phaneuf L & Fleming-Milici F (2022) Effects of sugary drink countermarketing videos on caregivers’ attitudes and intentions to serve fruit drinks and toddler milks to young children. Am J Public Health 112, S807–S816. 36288519 10.2105/AJPH.2022.307024 PMC9612202

[26] Morel K , Nichols K , Nong Y et al. (2019) Parental and provider perceptions of sugar-sweetened beverage interventions in the first 1000 days: a qualitative study. Acad Pediatr 19, 748–755. 30677540 10.1016/j.acap.2019.01.004 PMC6642844

[27] Taillie LS , Higgins ICA , Lazard AJ et al. (2022) Do sugar warning labels influence parents’ selection of a labeled snack for their children? A randomized trial in a virtual convenience store. Appetite 175, 106059. 35526703 10.1016/j.appet.2022.106059 PMC10173438

[28] McCann J , Woods J , Mohebbi M et al. (2022) Regulated nutrition claims increase perceived healthiness of an ultra-processed, discretionary toddler snack food and ultra-processed toddler milks: a discrete choice experiment. Appetite 174, 106044. 35430297 10.1016/j.appet.2022.106044

[29] Poirier B , Hedges J , Smithers L et al. (2021) ‘What are we doing to our babies’ teeth?’ Barriers to establishing oral health practices for Indigenous children in South Australia. BMC Oral Health 21, 1–12. 34488721 10.1186/s12903-021-01791-x PMC8422744

[30] Rhodes A (2022) Ready-Made Baby Foods: Do Parents Know the Facts? Royal National Children’s Hospital National Child Health Poll. https://rchpoll.org.au/wp-content/uploads/2022/04/NCHP24-Poll-report-A4_FA_WEB.pdf (accessed August 2024).

[31] Rowan M , Mirosa M , Heath ALM et al. (2022) A qualitative study of parental perceptions of baby food pouches: a netnographic analysis. Nutrients 14, 1–10. 10.3390/nu14153248 PMC9370201 35956424

[32] Sousa C (2022) Factors Contributing to Increase Brand Loyalty on Infant Nutrition Category in Portugal. NOVA Information Management School Instituto Superior de Estatística e Gestão de Informação. https://run.unl.pt/bitstream/10362/148931/1/TGI1630.pdf (accessed August 2024).

[33] UK Department of Health (2003) The Processed Cereal-Based Foods and Baby Foods for Infants the Processed Cereal-Based Foods and Baby Foods for Infants and Young Children (England) Regulations 2003. https://www.legislation.gov.uk/uksi/2003/3207/data.pdf (accessed July 2023).

[34] Adams J , Mytton O , White M et al. (2016) Why are some population interventions for diet and obesity more equitable and effective than others? The role of individual agency. PLoS Med 13, e1001990. 27046234 10.1371/journal.pmed.1001990 PMC4821622

[35] Garcia AL , Chee N , Vargas-Garcia EJ et al. (2024). Survey of emotional themes used in marketing of commercial baby foods in the UK—implications for nutrition promotion in early childhood. Int J Environ Res Public Health 21, 258. 38541260 10.3390/ijerph21030258 PMC10969795

[36] Euromonitor (2022) Children’s Food: A Playground of Opportunity. Market Research Report. https://www.euromonitor.com/childrens-food-a-playground-of-opportunity/report (accessed November 2024).

[37] Mintel (2023) Baby Food and Drink Market Report. Report Summary. https://store.mintel.com/report/uk-baby-food-and-drink-market-report (accessed November 2024).

