# Supplementary material for: How labelling of commercial infant food impacts parents’ beliefs about sugar content and related purchasing and feeding decisions: a scoping review
Source: Public Health Nutr. 2025 Aug 13;28(1):e138. doi: 10.1017/S1368980025100827 (PMC12516613; doi:10.1017/S1368980025100827)
Supplement: Conway et al. supplementary material 1 — Conway et al. supplementary material [file S1368980025100827sup001.docx]

**Scoping Review**

Search Strategies for: Medline, Embase, PsycINFO, CINAHL Plus, Web of Science (Core Collection), Cochrane Library.

**MEDLINE (Ovid) – searched 23/05**

| **Search** | **Terms** | **Records retrieved^1^** |
| --- | --- | --- |
| 1 | exp Parents/ or caregivers/ or grandparents/ | 197435 |
| 2 | (parent* or mother* or caregiver* or father* or grandparent* or grandmother* or grandfather* or guardian*).tw,kf | 843311 |
| 3 | 1 or 2 | 881170 |
| 4 | Infant food/ | 10285 |
| 5 | ((baby or babies or preschool* or toddler* or infan*) adj3 (food* or snack* or pouch* or jar or jars or meal* or beverag* or drink* or yog?urt* or cereal*)).tw,kf. | 5701 |
| 6 | 4 or 5 | 14191 |
| 7 | Beverages/ or Sugars/ or Dietary sugars/ or Sugar-sweetened Beverages/ | 24307 |
| 8 | ((Commercial* or packag* or sugar-sweet* or sugar*) adj3 (food* or snack* or pouch* or jar or jars or meal* or bevarag* or drink*)).tw,kf. | 21712 |
| 9 | (NAS or sugar* or sucrose*).tw,kf. | 237077 |
| 10 | Infant/ or Child, preschool/ or (baby or babies or preschool or pre-school or toddler* or infan* or child or children).tw,kf. | 2533448 |
| 11 | (7 or 8 or 9) and 10 | 15060 |
| 12 | 6 or 11 | 28231 |
| 13 | product packaging/ or exp food packaging/ or product labeling/ | 16302 |
| 14 | ((food* or sugar* or nutri* or health* or promotion* or traffic light* or TLL* or FOP* or front-of-pack* or product) adj3 (label* or warning* or information* or claim* or sign* or marketing or packag* or symbol* or sticker*)).tw,kf. | 196710 |
| 15 | 13 or 14 | 206516 |
| 16 | Consumer Behaviour/ or Choice Behavior/ | 35240 |
| 17 | (consumer* behavio?r* or choice behavio?r*).tw,kf. | 4505 |
| 18 | (knowledge* or understand* or choice* or influence* or perception* or perceive* or purchase* or opinion* or attitude* or view* or preference*).tw,kf. | 5427916 |
| 19 | 16 or 17 or 18 | 5436263 |
| 20 | 3 and 12 and 15 and 19 | 371 |
| 21 | limit 20 to English language | 326 |

**Embase (Ovid) – searched 23/05**

| **Search** | | **Terms** | **Records retrieved** |
| --- | --- | --- | --- |
| 1 | exp parent/ or caregiver/ or grandparent/ | | 403814 |
| 2 | (parent* or mother* or caregiver* or father* or grandparent* or grandmother* or grandfather* or guardian*).tw,kf | | 1071731 |
| 3 | 1 or 2 | | 1166082 |
| 4 | baby food/ or infant nutrition/ | | 5597 |
| 5 | ((baby or babies or preschool* or toddler* or infan*) adj3 (food* or snack* or pouch* or jar or jars or meal* or beverag* or drink* or yog?urt* or cereal*)).tw,kf. | | 7146 |
| 6 | 4 or 5 | | 10827 |
| 7 | artificially sweetened beverage/ or beverage/ or sweetened beverage/ or sugar/ or sugar-sweetened beverage/ or sugar industry | | 59399 |
| 8 | ((Commercial* or packag* or sugar-sweet* or sugar*) adj3 (food* or snack* or pouch* or jar or jars or meal* or beverag* or drink*)).tw,kf. | | 24499 |
| 9 | (NAS or sugar* or sucrose*).tw,kf. | | 263612 |
| 10 | Infant/ or preschool child/ or (baby or babies or preschool or pre-school or toddler* or infan* or child or children).tw,kf. | | 2788194 |
| 11 | (7 or 8 or 9) and 10 | | 19645 |
| 12 | 6 or 11 | | 37544 |
| 13 | packaging/ or food packaging/ or nutrition labeling/ | | 23685 |
| 14 | ((food* or sugar* or nutri* or health* or promotion* or traffic light* or TLL* or FOP* or front-of-pack* or product) adj3 (label* or warning* or information* or claim* or sign* or marketing or packag* or symbol* or sticker*)).tw,kf. | | 247508 |
| 15 | 13 or 14 | | 262452 |
| 16 | consumer attitude/ or purchasing/ or decision making | | 305522 |
| 17 | (consumer* behavio?r* or choice behavio?r*).tw,kf. | | 4508 |
| 18 | (knowledge* or understand* or choice* or influence* or perception* or perceive* or purchase* or opinion* or attitude* or view* or preference*).tw,kf. | | 5978890 |
| 19 | 16 or 17 or 18 | | 6162113 |
| 20 | 3 and 12 and 15 and 19 | | 512 |
| 21 | limit 20 to english language | | 489 |

**PsycINFO (Ovid) – searched 23/05**

| **Search** | | **Terms** | **Records retrieved** |
| --- | --- | --- | --- |
| 1 | exp parents/ or caregivers/ or grandparents/ | | 178134 |
| 2 | (parent* or mother* or caregiver* or father* or grandparent* or grandmother* or grandfather* or guardian*).tw,id | | 476753 |
| 3 | 1 or 2 | | 486439 |
| 4 | exp food/ | | 17852 |
| 5 | ((baby or babies or preschool* or toddler* or infan*) adj3 (food* or snack* or pouch* or jar or jars or meal* or beverag* or drink* or yog?urt* or cereal*)).tw,id | | 758 |
| 6 | 4 or 5 | | 18459 |
| 7 | exp Sugars/ or exp “Beverages (Nonalcoholic)”/ | | 12401 |
| 8 | ((Commercial* or packag* or sugar-sweet* or sugar*) adj3 (food* or snack* or pouch* or jar or jars or meal* or beverag* or drink*)).tw,id | | 2689 |
| 9 | (NAS or sugar* or sucrose*).tw | | 16916 |
| 10 | exp infant development/ or exp preschool student/ or (baby or babies or preschool or pre-school or toddler* or infan* or child or children).tw,id | | 794754 |
| 11 | (7 or 8 or 9) and 10 | | 2943 |
| 12 | 6 or 11 | | 20983 |
| 13 | exp product design/ or exp Warning Labels/ or Marketing/ | | 34217 |
| 14 | ((food* or sugar* or nutri* or health* or promotion* or traffic light* or TLL* or FOP* or front-of-pack* or product) adj3 (label* or warning* or information* or claim* or sign* or marketing or packag* or symbol* or sticker*)).tw,id | | 46369 |
| 15 | 13 or 14 | | 77722 |
| 16 | Consumer Attitudes/ or Choice Behavior/ or Eating Behavior/ or Consumer Behavior/ | | 81600 |
| 17 | (consumer* behavio?r* or choice behavio?r*).tw,id | | 15352 |
| 18 | (knowledge* or understand* or choice* or influence* or perception* or perceive* or purchase* or opinion* or attitude* or view* or preference*).tw,id | | 2035264 |
| 19 | 16 or 17 or 18 | | 2056675 |
| 20 | 3 and 12 and 15 and 19 | | 279 |
| 21 | limit 20 to english language | | 276 |

**CINAHL Plus (Ebsco) – searched 23/05**

| **Search** | | **Terms** | **Records retrieved** |
| --- | --- | --- | --- |
| S1 | (MH "Caregivers") | | 45225 |
| S2 | (MH “Grandparents”) | | 2581 |
| S3 | (MH “Parents+”) | | 122858 |
| S4 | TI ( parent* OR mother* OR caregiver* OR relative* OR father* OR grandparent* OR guardian* ) OR AB (parent* OR mother* OR caregiver* OR relative* OR father* OR grandparent* OR guardian*) | | 547499 |
| S5 | (MH “Infant Food”) | | 1562 |
| S6 | TI ( (baby or babies or preschool* or toddler* or infan*) N2 (food* or snack* or pouch* or jar or jars or meal* or beverag* or drink* or yoghurt* or cereal*) OR AB ( (baby or babies or preschool* or toddler* or infan*) N2 (food* or snack* or pouch* or jar or jars or meal* or beverage* or drink* or yoghurt* or cereal*) ) | | 1830 |
| S7 | S1 OR S2 OR S3 OR S4 | | 602253 |
| S8 | S5 OR S6 | | 3022 |
| S9 | (MH "Beverages") OR (MH "Sweetened Beverages") OR (MH "Fruit Juices+”) | | 9769 |
| S10 | (MH “Dietary Sucrose”) OR (MH “High Fructose Corn Syrup”) | | 5101 |
| S11 | TI ( (commercial* or packag* or sugar-sweet* or sugar*) n2 (food* or snack* or pouch* or jar or jars or meal* or beverag* or drink*) ) OR AB ( (Commercial or packag* or sugar-sweet* or sugar*) n2 (food* or snack* or pouch* or jar or jars or meal* or beverag* or drink*) ) | | 6535 |
| S12 | TI ( NAS or sugar* or sucrose*) OR AB ( NAS or sugar* or sucrose*) | | 31408 |
| S13 | (MH "Infant") OR (MH "Child, Preschool") | | 311317 |
| S14 | TI ( (baby or babies or pre-school or preschool or toddler* or infan* or child or children) ) OR AB ( ( baby or babies or preschool or pre-school or toddler* or infan* or child or children) ) | | 646469 |
| S15 | S9 OR S10 OR S11 OR S12 | | 41657 |
| S16 | S13 OR S14 | | 756270 |
| S17 | S15 AND S16 | | 7513 |
| S18 | S8 OR S17 | | 10159 |
| S19 | (MH "Product Packaging") OR (MH "Food Packaging”) OR (MH “Product Labeling”) | | 5559 |
| S20 | TI ( (food* or sugar* or nutri* or health* or promotion* or “traffic light*” or TLL* or FOP* or front-of-pack* or product) N2 (label* or warning* or information* or claim* or sign* or marketing or packag* or symbol* or sticker*) ) OR AB ( (food* or sugar* or nutri* or health* or promotion* or “traffic light*” or TLL* or FOP* or front-of-pack* or product) N2 (label* or warning* or information* or claim* or sign* or marketing or packag* or symbol* or sticker*) ) | | 72608 |
| S21 | S19 OR S20 | | 79926 |
| S22 | (MH "Consumer Attitudes") | | 9681 |
| S23 | (MH “Consumers”) | | 2985 |
| S24 | TI ( (knowledge* or understand* or choice* or influence* or perception* or perceive* or purchase* or opinion* or attitude* or view* or preference*) ) OR AB ( (knowledge* or understand* or choice* or influence* or perception* or perceive* or purchase* or opinion* or attitude* or view* or preference*) ) | | 1252105 |
| S25 | S22 OR S23 OR S24 | | 1258636 |
| S26 | S7 AND S18 AND S21 AND S25 | | 250 |
| S27 | limit S26 to english language | | 246 |

**Web of Science (Core Collection) – searched 23/05**

| **Search** | | **Terms** | **Records retrieved** |
| --- | --- | --- | --- |
| 1 | TS=(parent* OR caregiver* OR grandparent* OR mother* OR father* OR guardian*) | | 1282847 |
| 2 | TS=((baby or babies or preschool* or toddler* or infan*) NEAR/3 (food* or snack* or pouch* or jar or jars or meal* or beverag* or drink* or yoghurt* or yogurt* or cereal*)) | | 8841 |
| 3 | TS=((Commercial* or packag* or sugar-sweet* or sugar*) NEAR/3 (food* or snack* or pouch* or jar or jars or meal* or beverag* or drink*)) | | 45637 |
| 4 | TS=(NAS or sugar* or sucrose*) | | 465513 |
| 5 | TS=(baby or babies or preschool or pre-school or toddler* or infan* or child or children) | | 2767039 |
| 6 | **(#**3 OR **#**4) AND **#**5 | | 17047 |
| 7 | **#**2 OR **#**6 | | 24861 |
| 8 | **TS=((food* or sugar* or nutri* or health* or promotion* or “traffic light*” or TLL* or FOP*) NEAR/3 (label* or warning* or information* or claim* or sign* or marketing* or packag* or symbol* or sticker*))** | | 398261 |
| 9 | **TS=(("consumer behavio*”) OR ("choice behavio*”) OR ("consumer attitude*”))** | | 36670 |
| 10 | **TS=(knowledge* OR understand* OR choice* OR influenc* OR perception* OR perceive* OR purchas* OR opinion* OR attitude* OR view* OR preference*)** | | 12837276 |
| 11 | **#9 OR #10** | | 12842369 |
| 12 | **#1 AND #7 AND #8 AND #11** | | 528 |
| 13 | **#1 AND #7 AND #8 AND #11 and English (Languages)** | | 515 |

**Cochrane Library – searched 23/05**

| **Search** | | **Terms** | **Records retrieved** |
| --- | --- | --- | --- |
| 1 | [mh parents] | | 8720 |
| 2 | [mh ^caregivers] | | 3822 |
| 3 | [mh ^grandparents] | | 37 |
| 4 | (parent* or mother* or caregiver* or father* or grandparent* or grandmother* or grandfather* or guardian*):ti,ab,kw | | 97340 |
| 5 | [mh "Infant food"] | | 1869 |
| 6 | **((baby or babies or preschool* or toddler* or infan*) NEAR/3 (food* or snack* or pouch* or jar or jars or meal* or beverag* or drink* or yoghurt* or yogurt* or cereal*)):ti,ab,kw** | | 1832 |
| 7 | **[mh ^beverages]** | | 2214 |
| 8 | **[mh ^sugars]** | | 152 |
| 9 | **[mh ^"dietary sugars"]** | | 109 |
| 10 | **[mh ^"sugar sweetened beverages"]** | | 108 |
| 11 | **((Commercial* or packag* or sugar-sweet* or sugar*) NEAR/3 (food* or snack* or pouch* or jar or jars or meal* or beverag* or drink*)):ti,ab,kw** | | 2519 |
| 12 | **(NAS or sugar* or sucrose*):ti,ab,kw** | | 16832 |
| 13 | **[mh infant]** | | 46083 |
| 14 | **[mh "child, preschool"]** | | 39489 |
| 15 | **(baby or babies or preschool or pre-school or toddler* or infan* or child or children):ti,ab,kw** | | 232216 |
| 16 | **[mh "product packaging"]** | | 886 |
| 17 | **[mh "food packaging"]** | | 341 |
| 18 | **[mh ^"product labeling"]** | | 221 |
| 19 | **((food* or sugar* or nutri* or health* or promotion* or TLL* or FOP* or product) NEAR/3 (label* or warning* or information* or claim* or sign* or marketing or packag* or symbol* or sticker*)):ti,ab,kw** | | 20054 |
| 20 | **("traffic light" or "front of pack" or "front of package"):ti,ab,kw** | | 423 |
| 21 | **[mh ^"consumer behavior"]** | | 1176 |
| 22 | **[mh ^"choice behavior"]** | | 1783 |
| 23 | **("consumer behavior" or "choice behavior" or knowledge* or understand* or choice* or influence* or perception* or perceive* or purchase* or opinion* or attitude*):ti,ab,kw** | | 328643 |
| 23 | **#1 OR #2 OR #3 OR #4** | | 97340 |
| 25 | **#5 OR #6** | | 2629 |
| 26 | **#7 OR #8 OR #9 OR #10 OR #11 OR #12** | | 19233 |
| 27 | **#13 OR #14 OR #15** | | 232216 |
| 28 | **#26 AND #27** | | 3326 |
| 29 | **#28 OR #25** | | 5824 |
| 30 | **#16 OR #17 OR #18 OR #19 OR #20** | | 20469 |
| 31 | **#21 OR #22 OR #23** | | 328643 |
| 32 | **#24 AND #29 AND #30 AND #31** | | 144 |
|  | Cochrane doesn’t recommend language limitations | |  |
