# Supplementary material for: How labelling of commercial infant food impacts parents’ beliefs about sugar content and related purchasing and feeding decisions: a scoping review
Source: Public Health Nutr. 2025 Aug 13;28(1):e138. doi: 10.1017/S1368980025100827 (PMC12516613; doi:10.1017/S1368980025100827)
Supplement: Conway et al. supplementary material 2 — Conway et al. supplementary material [file S1368980025100827sup002.docx]

**HWPRU Scoping Review**

**Grey literature review**

**Search plan strategies:**

(1) Google search engines

(2) targeted websites, for example, Action on Sugar

(3) Contact experts, for example, Department of Health and Social Care

1. **Google Search engines strategy**

- Use incognito web browser to ensure search isn’t influenced by search history.
- Simplified search strings with multiple combinations of terms (see below)
- First 50 records on google and Google Scholar will be searched by one reviewer.
- Articles relevant at first glance (from title and blurb) will be included in next stage of screening process.
- Potentially relevant records recorded in an excel spreadsheet (google link, search string, article title)
- Full text screening conducted independently by two reviewers, with reasons for exclusion recorded in excel spreadsheet.

| Google &  Google Scholar  Search strings | **#1** “Sugar" AND "baby food" AND “label” AND "Parents" AND "Understanding"  **#2** “Sugar" AND "baby food" AND “warning labels” AND "Parents" AND "perspectives"  **#2** “Sugar" AND "baby food" AND “food label” AND "Parents" AND "perspectives" |
| --- | --- |
| Site-specific search strings for Google | **#1** site:gov.uk “warning labels” AND baby AND parent  **#2** site:gov.uk “sugar” AND baby AND parent  **#3** site:nhs.uk “warning labels” AND baby AND parent  **#4** site:gov.uk “sugar” AND baby AND parent  Smaller websites like First Steps Nutrition etc are easy to search manually so we probably don’t need to use a site-specific search strings. |

1. **Targeted websites**

- In addition to site-specific searches above, we will manually search key websites for anything that was missed using the site-specific Google search string “warning labels, baby and parent”
- First, we conducted a Google search to identify any relevant websites. A list of organisations/websites was complied.
- One reviewer will manually search each website for potentially relevant records.
- The full text of potentially relevant records will be added to excel and screened independently by two reviewers. Exclusion reasons will be recorded.

**Full list of websites to search:**

| **Organisation** | **Website** | **Date searched** |
| --- | --- | --- |
| Action on sugar | <https://www.actiononsugar.org/> |  |
| British Dental Association (BDA) | [www.bda.org](http://www.bda.org) |  |
| British Dietetic Association (BDA) | [www.bda.uk.com](http://www.bda.uk.com) |  |
| British Nutrition Foundation | [www.nutrition.org.uk](http://www.nutrition.org.uk) |  |
| Centers for Disease Control and Prevention (CDC) | [www.cdc.gov](http://www.cdc.gov) |  |
| Euromonitor | [www.Euromonitor.com](http://www.Euromonitor.com) |  |
| European Association for the Study of Obesity (EASO) | [www.easo.org](http://www.easo.org) |  |
| First Steps Nutrition | [www.firststepsnutrition.org/](http://www.firststepsnutrition.org/) |  |
| Food Active | [www.Foodactive.org.uk](http://www.Foodactive.org.uk) |  |
| Food Foundation | [www.foodfoundation.org.uk/](http://www.foodfoundation.org.uk/) |  |
| Food Regulation (AU/NZ) | [www.foodstandards.gov.au/](http://www.foodstandards.gov.au/) |  |
| Food Standards Agency (FSA) | [www.food.gov.uk/](http://www.food.gov.uk/) |  |
| Food Standards Australia and New Zealand (FSANZ) | [www.foodstandards.gov.au](http://www.foodstandards.gov.au) |  |
| Gov.uk |  |  |
| Infant and Toddler Forum | [www.infantandtoddlerforum.org/](http://www.infantandtoddlerforum.org/) |  |
| International Baby Food Action Network (IBFAN) | [www.ibfan.org/](http://www.ibfan.org/) |  |
| IGD Services | [www.Igd.com](http://www.Igd.com) |  |
| Mintel (needs thorough search) |  |  |
| National Institute for Health and Care Excellence (NICE) | [www.Nice.org.uk](http://www.Nice.org.uk) |  |
| Nesta | [www.nesta.org.uk](http://www.nesta.org.uk) |  |
| NHS.uk |  |  |
| Obesity Health Alliance (OHA) | [www.obesityhealthalliance.org.uk](http://www.obesityhealthalliance.org.uk) |  |
| Obesity UK | [www.obesityuk.org.uk/](http://www.obesityuk.org.uk/) |  |
| Organic Trade Association (OTA) | [www.ota.com/](http://www.ota.com/) |  |
| Royal College of Paediatrics and Child Health (RCPCH) | [www.rcpch.ac.uk/](http://www.rcpch.ac.uk/) |  |
| Royal Society for Public Health (RSPH) | [www.rsph.org.uk/](http://www.rsph.org.uk/) |  |
| Scientific Advisory Commission on Nutrition (SACN) | [www.gov.uk/government/groups/scientific-advisory-committee-on-nutrition](http://www.gov.uk/government/groups/scientific-advisory-committee-on-nutrition) |  |
| Sustain | [www.Sustainweb.org](http://www.Sustainweb.org) |  |
| The Association for the Study of Obesity (ASO) | [www.aso.org.uk](http://www.aso.org.uk) |  |
| UK Data Service | [www.ukdataservice.ac.uk/](http://www.ukdataservice.ac.uk/) |  |
| Urban Health | [www.Urbanhealth.org.uk](http://www.Urbanhealth.org.uk) |  |
| UNICEF UK | [www.unicef.org.uk/](http://www.unicef.org.uk/) |  |
| World Health Organisation (WHO) | [www.who.int/](http://www.who.int/) |  |
| World Obesity Federation | [www.worldobesity.org/](http://www.worldobesity.org/) |  |

**Reports already obtained through manual searching:**

| **Organisation** | **Report Author, Title & Date** |
| --- | --- |
| Action on Sugar | The sugar content of baby and toddler sweet snacks, and the health halo that surrounds them. 2021. Action on Sugar.  <https://www.actiononsugar.org/media/actiononsugar/Action-on-Sugar-Baby-&-Toddler-Sweet-Snacks-Report.pdf> |
| Action on Sugar | Sugar Awareness Week Report - Baby and toddler breakfasts. Action on Sugar. November 2022. <https://www.actiononsugar.org/media/actiononsugar/sugar-awareness-week/2022/Sugar-Awareness-Week-Report---Baby-&-Toddler-Breakfasts.pdf> |
| DHSC/PHE | Food and drinks aimed at infants and young children: evidence and opportunities for action. 2019. Dr Alison Tedstone, Jo Nicholas, Brittney MacKinlay, Bethany Knowles, Jeremy Burton, Gabrielle Owtram.  <https://www.actiononsugar.org/media/actiononsugar/sugar-awareness-week/2022/Sugar-Awareness-Week-Report---Baby-&-Toddler-Breakfasts.pdf> |
| Euromonitor | Children’s Food: A playground for opportunity. October 2022. (Limited content available free online, full report available for purchase) <https://www.euromonitor.com/childrens-food-a-playground-of-opportunity/report> |
| Food Standards Australia & New Zealand | Consumer Evidence Summary: No Added Sugar Claims’. 2022.  <https://www.foodstandards.gov.au/sites/default/files/2023-11/Consumer%20evidence%20summary%20no%20added%20sugar%20claims_final.pdf> |
| Mintel | UK Baby Food and Drink Market Report 2023 (Limited content available free online, full report available for purchase)  <https://store.mintel.com/report/uk-baby-food-and-drink-market-report> |
| Organic Trade Association | Consumer Perception of USDA Organic and Competing Label Claims Report. 2024. (Limited content available free online, full report available for purchase)  <https://ota.com/news/press-releases/19828> |
| Public Eye | Sugar: For Nestle, not all babies are equal  <https://www.publiceye.ch/en/media-corner/press-releases/detail/sugar-for-nestle-not-all-babies-are-equal> |
| WHO | Nutrient and promotion profile model: supporting appropriate promotion of food products for infants and young children 6–36 months in the WHO European Region. 2022.  <https://www.who.int/europe/publications/i/item/WHO-EURO-2022-6681-46447-67287> |

**3. Contact experts**

- Email individuals and organisations, including authors of relevant papers, and government and third sector organisations as listed above for website searches.
- Final responses due by 14/06/2024; all suggested reports will be added to excel
- Reports/articles relevant at first glance will be included in next stage of screening process.
- Full text screening conducted independently by two reviewers, with reasons for exclusion recorded in excel spreadsheet.
